# Supplementary material for: Predicting antigen specificity of single T cells based on TCR CDR3 regions
Source: Mol Syst Biol. 2020 Aug 11;16(8):e9416. doi: 10.15252/msb.20199416 (PMC7418512; doi:10.15252/msb.20199416)
Supplement: Supplementary file 1 — Appendix [file MSB-16-e9416-s001.pdf]

Appendix

Table of contents

Appendix Figure S1: The amino acid embedding choice does not strongly affect model performance. Distributions shown as boxplots are across threefold cross-validation.

Appendix Figure S2: Cellular doublet identification based on non-unique TCR chain reconstructions.

Appendix Figure S3: Number of unique TCR observations per antigen.

Appendix Figure S4: Categorical antigen models trained on single-cell data generalize to observations from IEDB and VDJdb that do not contain covariates present during training.

Appendix Figure S5: Models that embed antigen sequences to predict binding events cannot generalize well to unseen antigens.

Appendix Figure S6: Variation of TCR chain sequences by antigen specificity and donor in single-cell dataset.

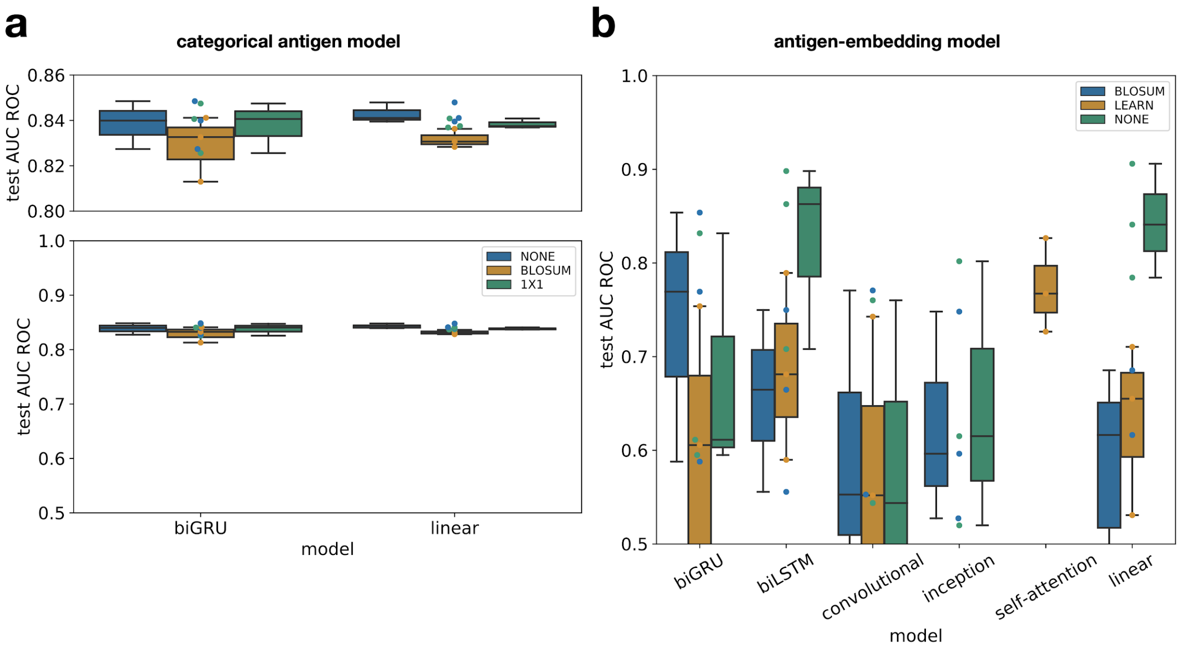

Appendix Figure S1: The amino acid embedding choice does not strongly affect model performance. Distributions shown as boxplots are across threefold cross-validation. (**a, b**) Comparison of model performance given multiple initial amino acid embeddings for models with antigen identity encoded in the output (**a**) and for models with sequence embedding of the antigen in the feature space (**b**). *BLOSUM*: BLOSUM52 embedding, *NONE*: one-hot encoding, *1X1*: five-dimensional 1×1 convolution on top of BLOSUM52 embedding that is learned at the time of training. All boxplots: the center of each boxplot is the sample median; the whiskers extend from the upper (lower) hinge to the largest (smallest) data point no further than 1.5 times the interquartile range from the upper (lower) hinge.

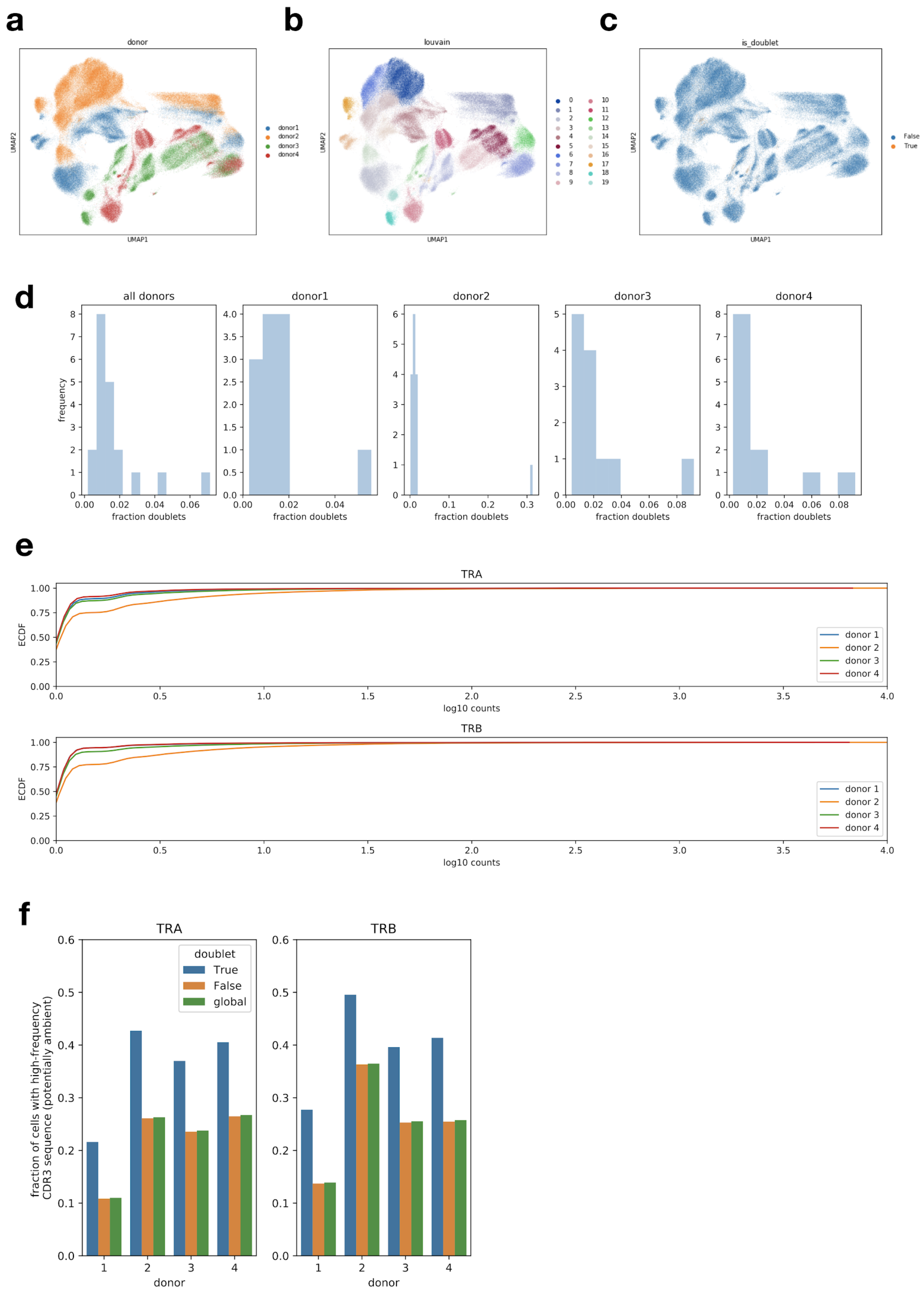

Appendix Figure S2: Cellular doublet identification based on non-unique TCR chain reconstructions. The discussion presented here is based on the conservative assumption that every cellular barcode shows more than one unique TCR allele for either the α- or the β-chain.

There are cells that have two active alleles for either chain but these cannot be easily separated from doublets that arise in the cell separation process. (**a–c**) UMAP of $CD8^+$ T cells from all donors (n=189,512) computed based on the transcriptome with (**a**) donor identity, (**b**) louvain cluster and (**c**) inferred doublet state superimposed. (**d**) Distribution of fractions of doublet out of all cells per clustering computed for each donor and for all clustering computed across all donors. (**e**) Empirical cumulative density function (ECDF) of the number of T cells that have a given CDR3 TCR sequence by chain and donor. “log10 counts” on the x-axis are the base 10 logarithm of the number of T cells for a given CDR3 sequence. (**f**) The fraction of cells that contain high-frequency CDR3 sequences that occur in more than 50 clonotypes. These high-frequency sequences are defined separately for each donor and may partially represent sequences derived from ambient molecules (Methods and Protocols). *True*: is doublet, *False*: is not doublet, *global*: all cells, doublets, and non-doublets.

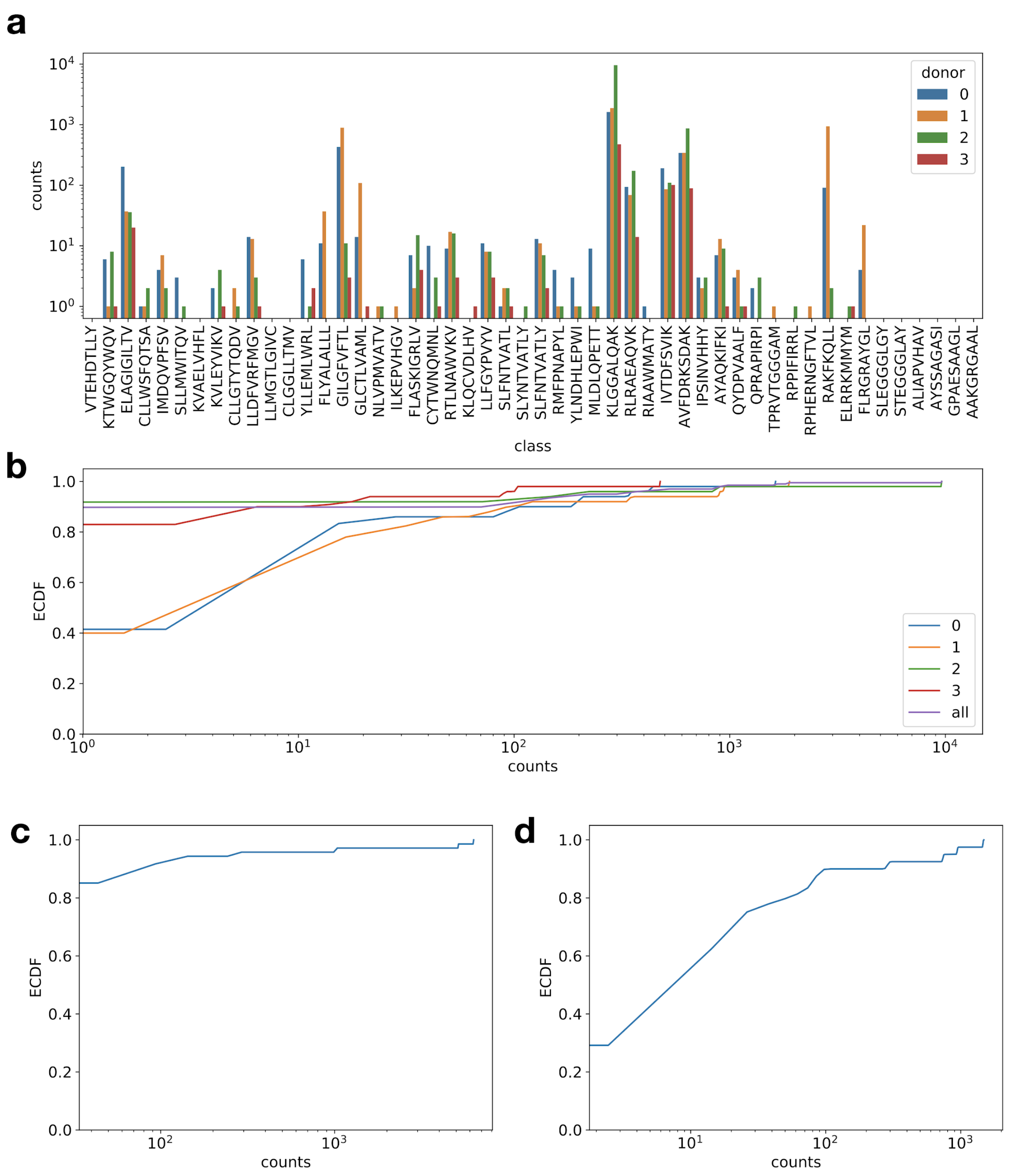

Appendix Figure S3: Number of unique TCR observations per antigen. (**a**) Histogram with the number of TCR clonotypes by antigen and donor for single-cell immune repertoire data. (**b–d**) Empirical cumulative density function (ECDF) of number of clonotypes (counts) per antigen for single-cell immune repertoire data (**b**), IEDB (**c**), and VDJdb (**d**).

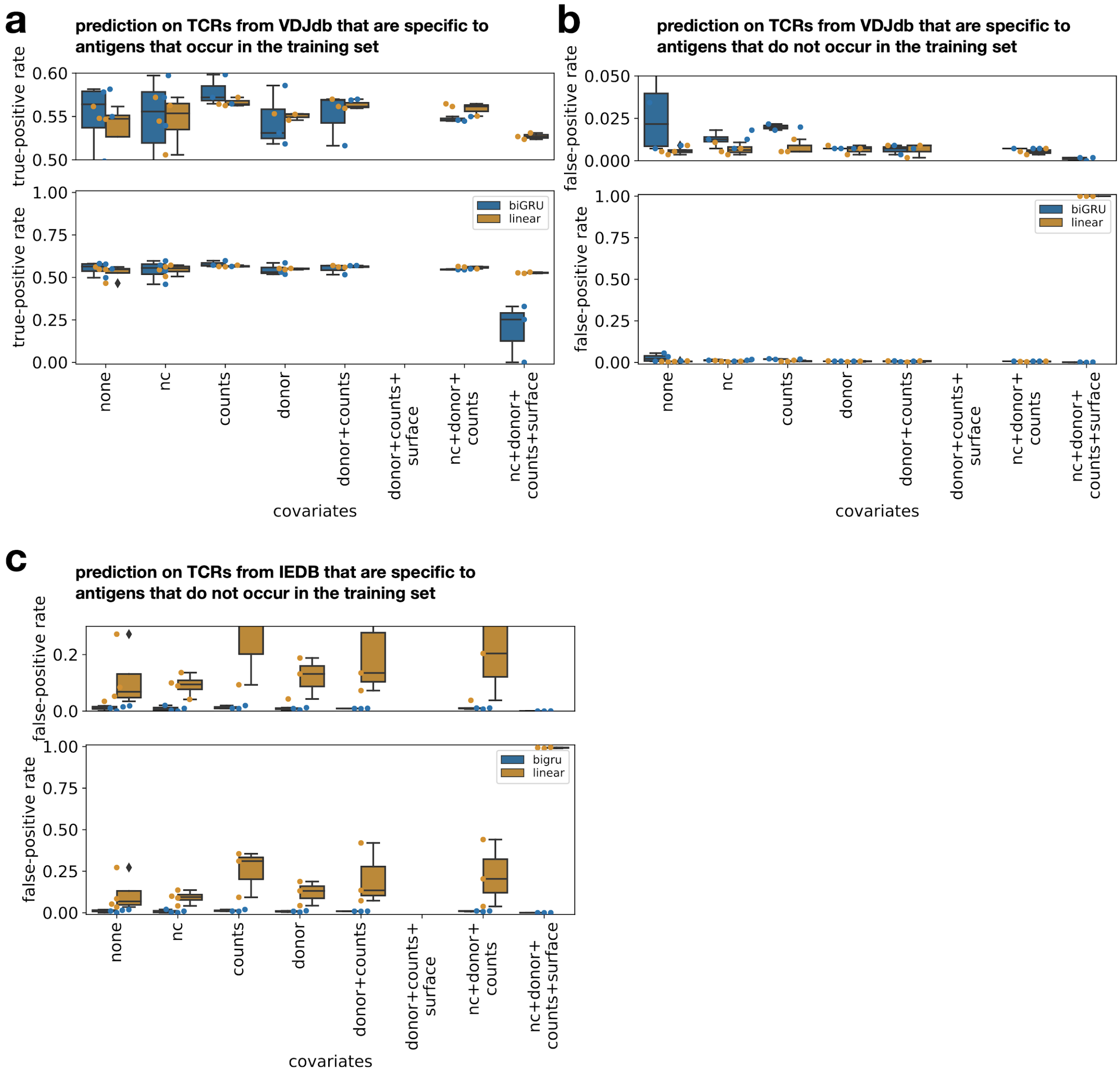

Appendix Figure S4: Categorical antigen models trained on single-cell data generalize to observations from IEDB and VDJdb that do not contain covariates present during training. Distributions shown as boxplots are across threefold cross-validation. (**a**) True-positive rate of the best-performing model by layer type and covariate setting on VDJdb entries with antigens that occur in the pMHC panel. All observations in this set should be predicted as positive for one of the categories of the model. *counts*: total mRNA counts, *nc*: negative-control pMHC counts, *surface*: surface protein counts. (**b, c**) The false-positive rate of best-performing model by layer type and covariate setting on VDJdb (**b**) and IEDB (**c**) entries with antigens that do not occur in the pMHC panel. All observations in this set should be predicted as negative (not binding any antigen of the panel). All boxplots: the center of each boxplot is the sample median; the whiskers extend from the upper (lower) hinge to the largest (smallest) data point no further than 1.5 times the interquartile range from the upper (lower) hinge.

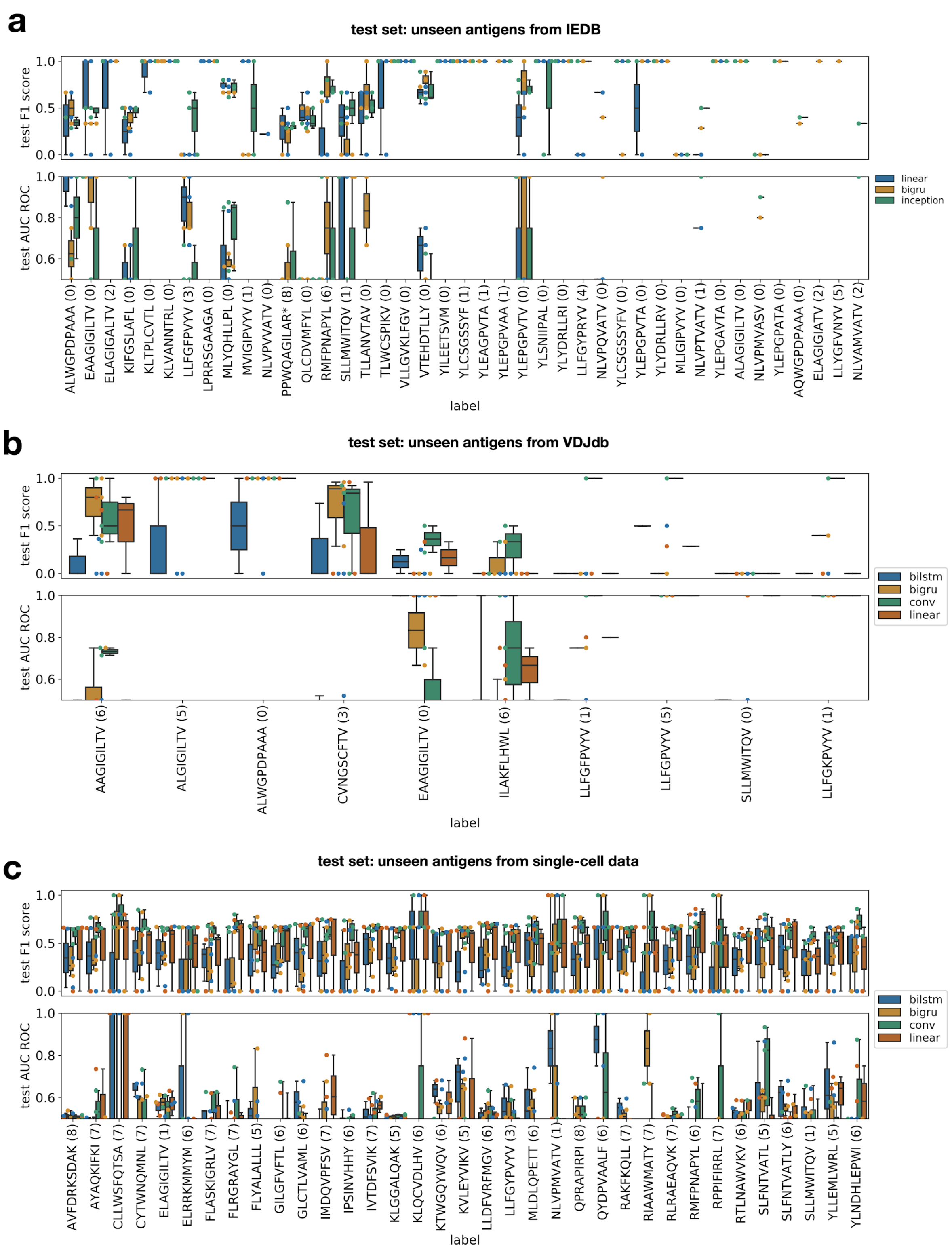

Appendix Figure S5: Models that embed antigen sequences to predict binding events cannot generalize well to unseen antigens. All models shown were trained on observations from IEDB and were tested on unseen low-frequency antigens from IEDB **(a)**, unseen antigens from VDJdb **(b),** or unseen antigens from the single-cell data set **(c)**. *BIGRU*: models trained with bidirectional GRUs as sequence-embedding layers. *NETTCR*: NetTCR-like model. *LINEAR*:

models trained with a single densely connected layer as a sequence-embedding layer. *test AUC ROC*: area under the receiver operator characteristic curve on the test set for the binary binding event prediction task, *F1 score*: F1 score on binary predictions on the test set. Distributions shown as boxplots are across threefold cross-validation. All boxplots: the center of each boxplot is the sample median; the whiskers extend from the upper (lower) hinge to the largest (smallest) data point no further than 1.5 times the interquartile range from the upper (lower) hinge.

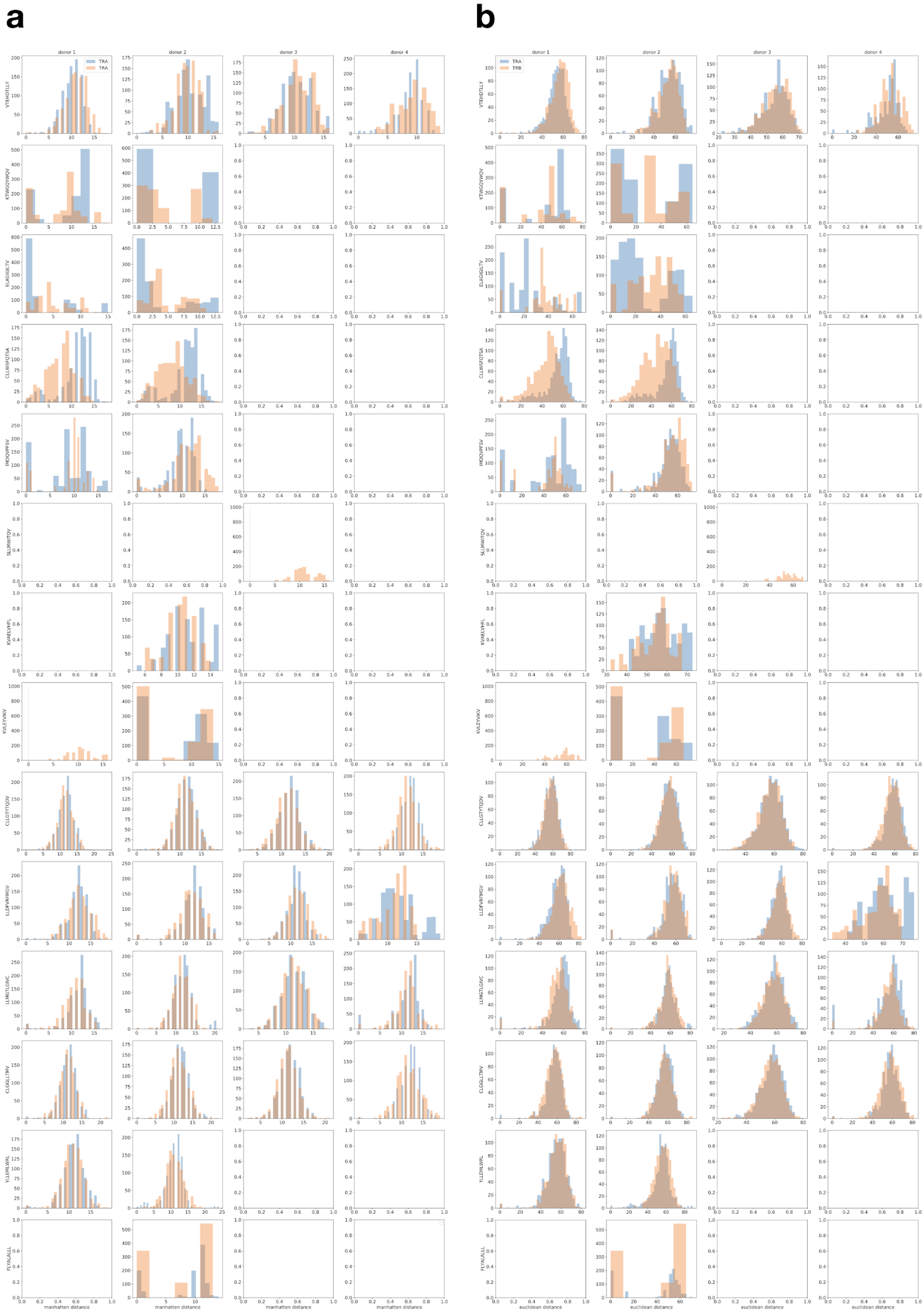
a
b
donor 1
donor 2
donor 3
donor 4
TRA
TRA
TRB
VTEHDTLLY
KTWGQYWQV
ELAGIGILTV
CLLWSFQTSA
IMDQVPFSV
SLLMWITQV
KVAELVHFL
KVLEYVIKV
CLLGTYTQDV
LLDFVRFMGV
LLMGTLGIVC
CLGGLLTMV
YLLEMLWRL
FLYALALLL
manhatten distance
euclidean distance

Appendix Figure S6: Variation of TCR chain sequences by antigen specificity and donor in single-cell dataset. Shown is the distribution of pairwise distances between randomly sampled pairs of cells with a common antigen specificity for each donor. For each plot, first putative doublets were excluded (Materials and Methods) and clonotypes within donors subsampled to a maximum of 10 cells. Each empirical distribution represents n=10,000 sampled pairs. In **(a)**, distance is evaluated as Manhattan distance on one-hot encoded sequences. In **(b)**, distance is evaluated as euclidean distance on BLOSUM50 encoded sequences. Combinations of donor and antigen with less than 10 putative non-doublet cells are excluded in this plot and appear as white squares.
